# Radiomics-based machine learning in the prediction of peritoneal metastasis in ovarian cancer: a systematic review and meta-analysis

**DOI:** 10.1186/s12880-025-02068-3

**Published:** 2025-12-02

**Authors:** Mohsen Salimi, Pouria Vadipour, Ali Abdolizadeh, Farzad Fayedeh, Sharareh Seifi

**Affiliations:** 1 Research Center of Thoracic Oncology (RCTO), National Research Institute of Tuberculosis and Lung Diseases (NRITLD), Shahid Beheshti University of Medical Sciences, Tehran, Iran; 2 Western Michigan University Homer Stryker M.D. School of Medicine, Kalamazoo, MI USA; 3 Anatomical Sciences, School of Medicine, St. George’s University, St. George’s, Grenada; 4 Student Research Committee, Birjand University of Medical Sciences, Birjand, Iran

**Keywords:** Radiomics, Machine learning, Ovarian cancer, Peritoneal metastasis, Artificial intelligence, Peritoneal carcinomatosis

## Abstract

**Background and purpose:**

Peritoneal metastasis significantly impacts prognosis and treatment strategies in ovarian cancer. Traditional imaging techniques have limited sensitivity in preoperative detection. Radiomics-based machine learning models offer a promising non-invasive approach to improve diagnostic accuracy. This study systematically reviews and meta-analyzes their predictive performance.

**Materials and methods:**

A systematic search was conducted up to June 2025 for studies that developed and validated machine learning models based on radiomic features for the prediction of peritoneal metastasis in ovarian cancer. Quality of included studies was evaluated using the METRICS and QUADAS-2 tool. Pooled sensitivity, specificity, and area under the curve (AUC) were calculated via bivariate random-effects meta-analysis. Heterogeneity, sensitivity, and publication bias analyses were also conducted.

**Results:**

Six studies were included in the systematic review and qualitative synthesis, with five studies comprising 448 individual participants derived exclusively from validation cohorts meeting criteria for meta-analysis. Radiomics models yielded a pooled AUC of 0.81 (95% CI: 0.71–0.88), accompanied by a sensitivity of 73% and specificity of 77%. Clinical models demonstrated a pooled AUC of 0.82 (95% CI: 0.79–0.85). The pooled AUC for the combined clinical–radiomics models was 0.87 (95% CI: 0.83–0.89). Methodological quality assessed via METRICS ranged from 64.5% to 86.3%, with a mean score of 77.6%. The QUADAS-2 assessment indicated a low overall risk of bias, with minor concerns noted in the patient selection and index test domains. No significant heterogeneity, publication bias, or outliers were detected.

**Conclusion:**

Radiomics-based machine learning models demonstrate potential as tools for the prediction of peritoneal metastasis in ovarian cancer and may assist in preoperative risk stratification. Further large-scale, multicenter prospective studies with standardized methodologies and external validation are necessary to confirm clinical applicability.

**Supplementary Information:**

The online version contains supplementary material available at 10.1186/s12880-025-02068-3.

## Introduction

Ovarian cancer (OC) constitutes a major global health challenge, ranking as the fifth most frequently diagnosed malignancy among women worldwide and the most lethal of all gynecologic malignancies [1]. In the United States, OC remains the primary cause of mortality among gynecologic malignancies and the sixth most common cause of cancer-related mortality in women [2]. Despite therapeutic advances, prognosis remains poor, particularly in advanced-stage disease, where 5-year survival rates range from just 32% to 47% [3]. Among OC subtypes, epithelial ovarian cancer (EOC) accounts for over 90% of all cases and is the primary driver of mortality. A hallmark of EOC is its tendency for early and widespread intraperitoneal dissemination, with nearly 75% of patients diagnosed at an advanced stage [4]. This often includes peritoneal metastasis (PM), particularly extra pelvic peritoneal metastasis (EPM), defined as tumor spread beyond the pelvic brim. EPM is a key determinant of advanced-stage disease, In accordance with the International Federation of Gynecology and Obstetrics staging system (FIGO) staging [5].

PM typically manifests as numerous small, superficial implants scattered across peritoneal surfaces and abdominal organs, complicating detection and limiting curative surgical outcomes [6]. Accurate preoperative assessment of peritoneal involvement is therefore essential for guiding clinical decision-making. It informs treatment planning, including the need for neoadjuvant chemotherapy (NACT), referral strategies, biopsy targeting, and surgical approach [7, 8]. Because PM has direct implications for prognosis and operability, improving its preoperative prediction is critical to optimizing outcomes in patients with advanced-stage EOC.

Radiological imaging plays an essential role in the preoperative assessment and staging of EOC, particularly in evaluating PM [9]. Among the imaging modalities, computed tomography (CT) remains the most widely used non-invasive technique for staging and for spread assessment. Multidetector CT is often the modality of choice for tumor–node–metastasis staging and for evaluating the extent of disease at recurrence [10]. However, CT has notable limitations, including poor soft-tissue contrast and limited sensitivity for detecting small or isodense implants, especially those located in anatomically complex regions such as the mesenteric root or small bowel serosa [11, 12]. Diagnostic accuracy is particularly low in patients with occult peritoneal metastases, which are metastatic deposits not visible on CT but later confirmed via surgery. This challenges radiologists in the absence of typical imaging findings such as omental caking or ascites [13].

Magnetic resonance imaging (MRI), notably with diffusion-weighted imaging (DWI), has emerged as a highly accurate alternative due to its superior soft-tissue resolution. MRI has demonstrated improved detection of small peritoneal metastases, even without ascites, because of its ability to highlight hypercellular implants on a hypointense background [14, 15]. MRI is particularly valuable in assessing operability and predicting the peritoneal cancer index, especially when CT findings are inconclusive [16]. Nevertheless, the interpretation of MRI can be subjective and highly dependent on reader’s expertise, limiting its reliability in detecting subtle metastatic deposits [17].

Ultrasound (US) remains a commonly used, accessible, and non-invasive imaging modality, particularly in the initial assessment of ovarian tumors. It is also valuable in distinguishing between benign and malignant adnexal masses and in early screening contexts. Although the US can detect features such as ascites, peritoneal nodules, and mass relationships to surrounding tissues, its utility in detecting peritoneal metastases is limited. The accuracy of US is affected by factors such as tumor location, size, and patient body habitus. As a result, US is generally used in combination with cross-sectional imaging for diagnostic work-up and surveillance [18, 19].

In response to these limitations, radiomics has emerged as a promising strategy to augment the diagnostic value of existing imaging. Radiomics refers to the high-throughput extraction and analysis of quantitative features from conventional medical images, capturing tumor heterogeneity, morphology, and texture in ways not discernible to the human eye [20]. In OC, radiomics-based models derived from CT, MRI, and US have shown promise in improving preoperative prediction of PM [19, 21].

Despite these advances, the current body of literature remains highly heterogeneous. Studies vary considerably in imaging modalities, acquisition protocols, segmentation methods, feature extraction pipelines, machine learning (ML) algorithms, and validation strategies. Although many radiomics models report promising sensitivity and specificity, concerns persist regarding reproducibility, robustness, and clinical generalizability. The absence of standardized workflows and reporting guidelines hampers cross-study comparisons and limits the ability to draw firm conclusions. To address these gaps, we conducted a systematic review and meta-analysis to critically assess the diagnostic performance and methodological quality of radiomics-based models for the preoperative prediction of PM in EOC. Our goal is to synthesize current evidence, evaluate methodological quality, and inform future efforts aimed at clinical translation.

## Materials and methods

This study’s protocol was registered with the Prospective Register of Systematic Reviews under registration number CRD420251088315. This systematic review and meta-analysis was conducted in accordance with the Preferred Reporting Items for a Systematic Review and Meta-analysis of Diagnostic Test Accuracy Studies (PRISMA-DTA) guidelines [22].

### Literature search strategy

A comprehensive literature search was undertaken through four databases (PubMed, Scopus, Embase, Web of Science) until June 18, 2025. The search strategy was initially developed by one author and subsequently reviewed and approved by a second author to ensure its accuracy and comprehensiveness. The search used terms related to the study topic (e.g., ‘peritoneal metastasis’, ‘ovarian’, and ‘radiomics’) along with Boolean operators (AND, OR) and wildcards to account for spelling and terminology differences. Table S1 contains the full search queries for each database. In addition to the database search, the reference lists of included studies and relevant previously published systematic reviews were manually examined to identify any additional pertinent articles. The search did not specify the language or publication year of the manuscript.

### Study selection

Search result records were imported into EndNote 21.0. Initially, the records were manually screened for duplicates. After removing duplicates, the remaining records underwent title and abstract screening to select relevant studies. Finally, these records underwent full-text screening to determine which studies to include in our review. The inclusion criteria for the studies were as follows: (1) studies involving patients diagnosed with OC, (2) studies predicting PM in OC patients, (3) studies that developed ml models for this prediction, (4) studies using radiomics features extracted from imaging modalities as input for the ml models, and (5) studies reporting model performance using metrics such as area under the curve (AUC), sensitivity, and specificity. The exclusion criteria were as follows: (1) review articles, conference abstracts, book chapters, and case reports; (2) studies with outcomes that did not match the focus of this review; and (3) studies that did not classify patients based on predicting PM but performed other tasks, such as segmentation.

For inclusion in the meta-analysis, studies had to meet the following criteria: (1) present sufficient data to construct a 2 × 2 confusion matrix; (2) validate their model on a separate cohort from the one used for training; and (3) have no overlap in training cohorts with other included studies. If cohort overlap was identified, the study with the larger sample size was included; if the sample sizes were the same, the most recent study was selected. The entire study selection process was performed independently by two authors, and any disagreements were resolved through discussion with a third author.

### Data extraction

Data extraction was carried out independently by two reviewers and later cross-verified during the final stage of the process. Disagreements were resolved by referral to a third author. The extracted information from the included studies encompassed several categories: general study details, population characteristics, and specifics related to the index tests. These index test details included methods used, algorithms, validation types, segmentation techniques, feature selection strategies, preprocessing steps, software for segmentation, and feature extraction. If any non-English manuscript had met the inclusion criteria, its full text and supplementary materials would have been translated into English prior to data extraction.

To rigorously evaluate the extent to which the results are generalizable, only metrics derived from independent validation populations—comprising subjects distinct from those in the training sets—were incorporated. In situations where studies reported both internal and external validation findings, preference was given exclusively to external validation outcomes for subsequent analysis. Furthermore, to assess the practical application of radiomic characteristics, data were extracted from validation cohorts in investigations that constructed predictive frameworks utilizing either clinical indicators (such as ascitic fluid presence or CA125 serum levels), radiomic variables, or a synthesis of both clinical and radiomic inputs. The diagnostic capabilities of radiomic-only, clinical-only, and combined clinical-radiomic approaches were aggregated and examined separately through three distinct evaluative processes. When multiple radiomic approaches were available within a single study, the one demonstrating superior discriminatory power—measured by the AUC in the validation cohort—was selected for inclusion. For definitional clarity, clinical approaches refer to those developed exclusively from patient clinical data, whereas clinical-radiomic approaches incorporate both clinical parameters and quantitative imaging features.

For each included study, 2 × 2 contingency tables were reconstructed using the reported sensitivity, specificity, and the number of metastatic (positive) and non-metastatic (negative) patients. If a study explicitly defined non-metastatic cases as positive and metastatic cases as negative, we reversed the coding to ensure consistency in positive/negative classification across all studies.

Specifically,

- True positive = number of metastatic × sensitivity.
- False negative = number of metastatic × (1 − sensitivity).
- True negative = number of non-metastatic × specificity.
- False positive = number of non-metastatic cases × (1 − specificity).

When sensitivity and specificity were not explicitly reported, we applied the top-left (Youden index) method [23] to determine the optimal cut-off point and derived sensitivity and specificity from that threshold to reconstruct the 2 × 2 matrix.

### Quality assessment

To evaluate the quality of the included studies, two primary assessment tools were utilized: QUADAS-2 [24] and the Methodological Radiomics Score (METRICS) [25]. These instruments were selected to capture different dimensions of methodological rigor pertinent to the aims of this review. Quality assessment was conducted independently by two reviewers, with discrepancies resolved through consensus or consultation with a third reviewer.

The QUADAS-2 tool was adapted to fit the specific context of this review. Two independent reviewers modified the original questions to better reflect the scope of this analysis, and any differences in interpretation were addressed collaboratively. A detailed description of the QUADAS-2 evaluation is provided in the supplementary materials (Table S2). Visual representations of the results were generated using RevMan 5 software.

In parallel, the METRICS tool was applied to assess methodological quality across nine core domains. The complete set of evaluation items and detailed descriptions can be accessed at: https://metricsscore.github.io/metrics/METRICS.html. Overall, the use of both QUADAS-2 and METRICS allowed for a comprehensive appraisal of study quality from both clinical and technical perspectives.

### Statistical analysis

The meta-analysis was performed using the MIDAS module in STATA (version 15), applying a bivariate random-effects model to compute the pooled estimates of sensitivity, specificity, and AUC, along with their respective 95% confidence intervals. To visualize diagnostic accuracy, individual study sensitivity and specificity estimates were plotted on the ROC space, together with a Summary Receiver Operating Characteristic (SROC) curve generated from the bivariate random-effects model. To evaluate variability across studies, heterogeneity was assessed using both the Cochran’s Q test and the I² statistic. A Cochran Q test p-value below 0.05, combined with an I² exceeding 50%, was interpreted as indicating substantial heterogeneity [26]. To explore potential contributors to this heterogeneity, subgroup analyses were performed based on various covariates, including study design, number of centers, type of validation, ML algorithm, sample size, METRICS score, imaging modality, and segmentation software used. The robustness of the results was further examined through sensitivity analyses, which involved detecting and evaluating outliers, as well as performing influence diagnostics. To detect potential publication bias, Deeks’ funnel plot asymmetry test was applied, with statistical significance set at *p* < 0.05 [27]. Several visualizations used throughout the analysis, including plots, were created using Python 3.10 with the matplotlib library.

## Results

### Systematic search and study selection

**Fig. 1:**
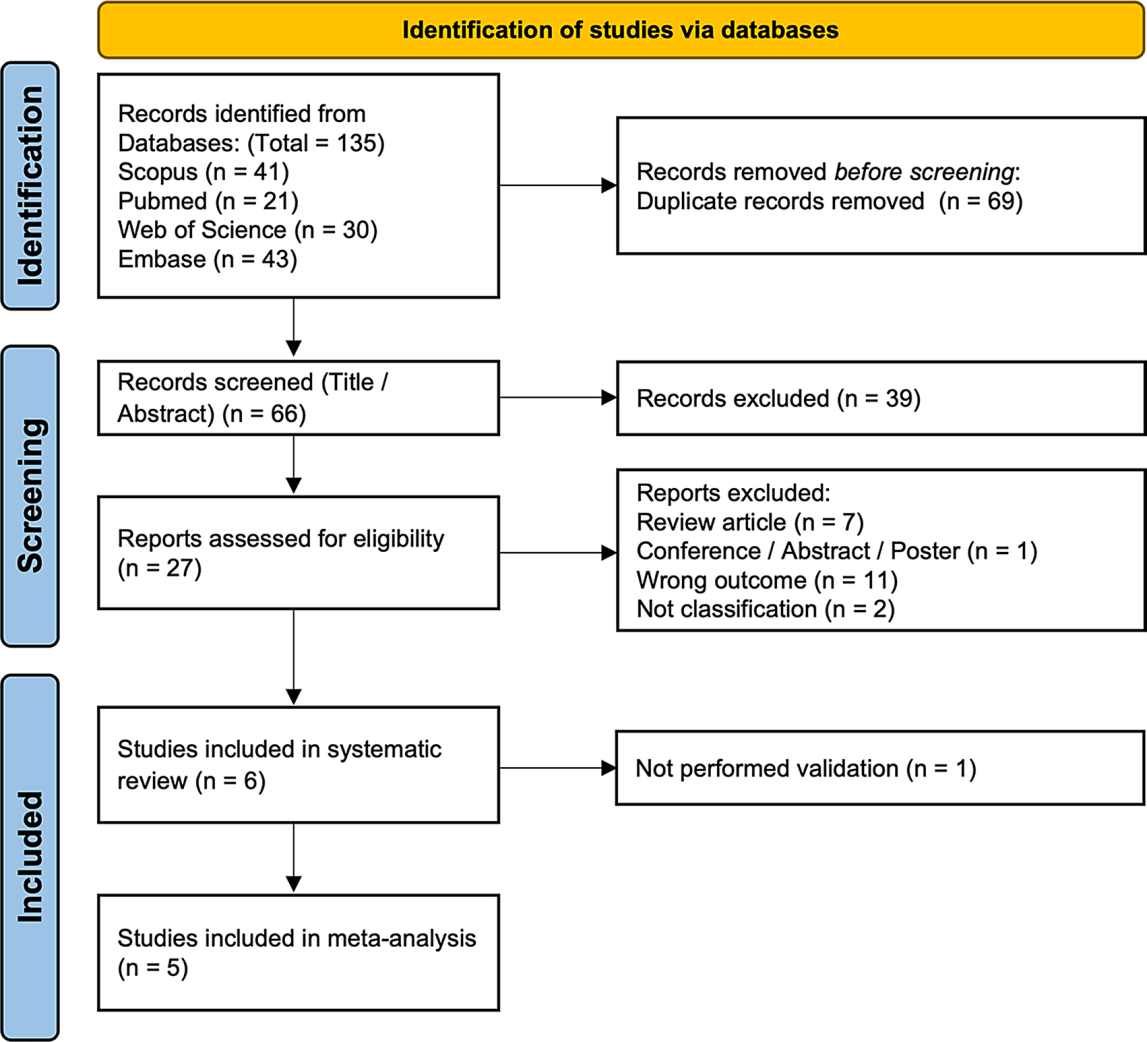
PRISMA flow diagram for the systematic review and meta-analysis

A total of 135 records were identified through database searches. After removing 69 duplicate records before screening, 66 records remained for title and abstract screening. Out of the 66 screened records, 39 were excluded based on irrelevance to the study topic. The remaining 27 full-text articles were assessed for eligibility. Ultimately, 6 studies met the inclusion criteria and were included in the systematic review (all in English language) [19, 21, 28–31]. However, one study was excluded from the meta-analysis due to the lack of validation. Therefore, a total of 5 studies were included in the final meta-analysis [19, 28–31] (Fig. 1). The general characteristics and methodological details of the radiomics and ML workflows in the included studies are summarized in Table 1.

**Table 1:** General characteristics and methodological details of the radiomics and machine learning workflow in included studies

Study	Country	Design	Modality	Segmentation	Annotator(s)	Segmentation/FE Software	Segmentation Validation	Preprocessing	RF Extracted/Selected	Feature Selection	Validation	ML Algorithm	CV	Train/IV/EV Size	Clinical Indicators Used in Combined Models
Zhou, Y. 2025	China	R/MC	US	Manual/3D	2 Independent Radiologist	3D Slicer/PyRadiomics	IRA, IOA	Applied	1049/7	LASSO	Sample Split, External	WLC by LASSO coefficients	10-fold-CV	374/164/153	Ascites, Fallopian tube invasion, Greatest diameter, HE4, D-dimer level
Wei, M. 2024	China	R/MC	MRI (T2WI)	Manual/3D	1 Radiologist	ITK-SNAP/PyRadiomics	NA	Applied	1223/15	Mann Whitney U test, SCC, mRMR, LASSO	Sample Split, External	SVM	NA	297/75/53 + 54	Parity, Abdominal Distention, CA125, HE4, and CA199
Li, J. 2024	China	R/SC	CT (CECT)	Manual/3D	2 Independent Radiologist	3D Slicer/PyRadiomics	IRA, IOA	Applied	1316/14	PCC, SCC, LASSO	Sample Split	WLC by LASSO coefficients	10-fold-CV	156/68/-	HE-4, CA-125, Laterality of lesion, Thickened septa, Margin
Wang, X. 2024	China	R/MC	MRI (T2WI)	Manual/3D	2 Independent Radiologist	ITK-SNAP/PyRadiomics	IRA, IOA	Applied	1130/13	Mann Whitney U test, SCC, LASSO	Sample Split, External	SVM	10-fold-CV	245/105/138	CA125, HE4, Parity, Abdominal pain
Yu, X. Y. 2021	China	R/SC	MRI (FS-T2WI, DWI, DCE-MRI)	Manual/3D	2 Independent Radiologist	3D Slicer/PyRadiomics	IRA, IOA	Applied	3111/6	mRMR, LASSO	cross-validation	LR	10-fold-CV	86/-/-	CA125 level
Song, X. L. 2021	China	P/SC	MRI (FS-T2WI, T2WI, DWI)	Manual/3D	2 Independent Radiologist	ITK-SNAP/PyRadiomics	IOA, Dice Coefficient (DC)	Applied	3111/7	Wilcoxon signed-rank test, LASSO, AIC	Sample Split	LR	3-fold-CV	54/35/-	Age, CA-125 level, CA-153 level, Pelvic fluid

R: Retrospective, SC: Single-center, MC: Multi-center, US: Ultra Sonography, MRI: Magnetic Resonance Imaging, CT: Computed Tomography, CECT: Contrast-Enhanced Computed Tomography, T2WI: T2-Weighted Imaging, FS-T2WI: Fat‑Suppressed T2-Weighted Imaging, DWI: Diffusion-Weighted Imaging, DCE-MRI: Dynamic Contrast-Enhanced Magnetic Resonance Imaging, NA: Not Applied, IRA: Intra-observer Agreement, IOA: Inter-observer Agreement, ML: Machine Learning, FE: Feature Extraction, RF: Radiomics Features, LASSO: Least Absolute Shrinkage and Selection Operator, PCC: Pearson Correlation Coefficient, SCC: Spearman Correlation Coefficient, mRMR: Minimum Redundancy Maximum Relevance, LR: Logistic Regression, CV: Cross-Validation, WLC: Weighed Linear Combination, SVM: Support Vector Machine, IV: Internal Validation, EV: External Validation

### Quality assessment

#### QUADAS-2

**Fig. 2:**
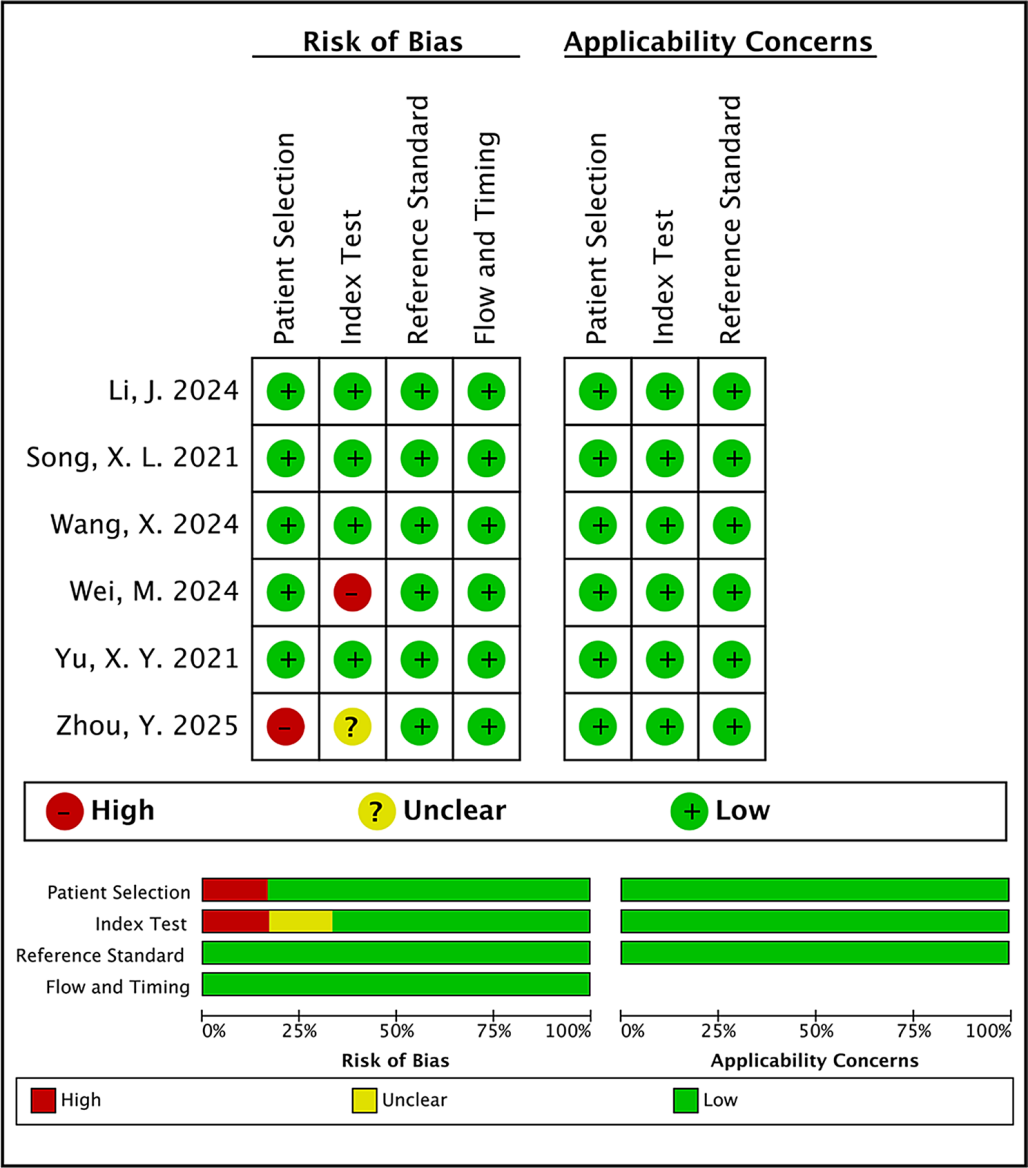
Quality assessment of included studies showing risk of bias and applicability concerns based on QUADAS-2

The QUADAS-2 assessment yielded the following findings related to the risk of bias within the included studies: 1/6 (16.7%) had a high risk of bias in patient selection, while 1/6 (16.7%) demonstrated high and 1/6 (16.7%) Demonstrated an unclear risk of bias in the index test domain, largely attributable to inadequate segmentation validation and inconsistent imaging protocols. No study had a high or unclear risk of bias in the reference standard, and flow and timing domain. No notable concerns were observed in the applicability assessment. Overall, the studies exhibited minimal methodological limitations, with concerns primarily confined to the index test and patient selection domains (Fig. 2) (Table S3).

#### METRICS

**Fig. 3:**
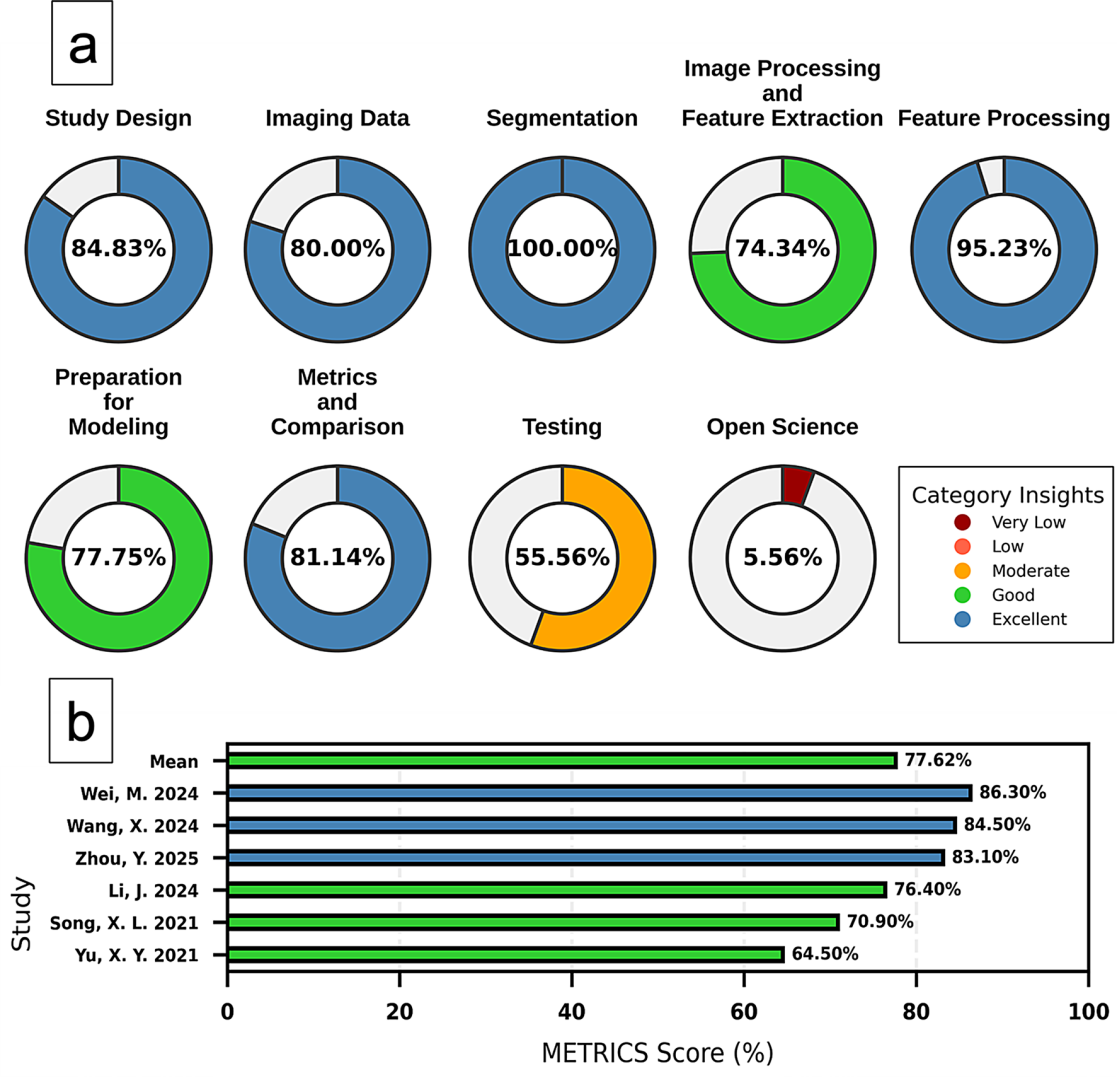
Methodological quality assessment using METRICS. (**a**) Weighted average METRICS score for each domain. (**b**) METRICS scores across individual studies and the overall mean score. Colors correspond to percentage categories of METRICS scores as indicated in the legend

The METRICS percentage of studies ranged from 64.50 to 86.30%, with a mean of 77.62% overall. Among the included studies, 3/6 (50%) was categorized as excellent, and 3/6 (50%) as good (Fig. 3). A comprehensive description of the METRICS scores, along with detailed domain-specific assessments, is provided in the supplementary materials (Table S4).

### Meta-analysis

#### Diagnostic test accuracy analysis

This meta-analysis included 5 studies with a total of 498 participants from validation cohorts. Each study developed three separate models: a radiomics model, a clinical model, and a combined clinical-radiomics model. These models were pooled separately in distinct analyses.

**Fig. 4:**
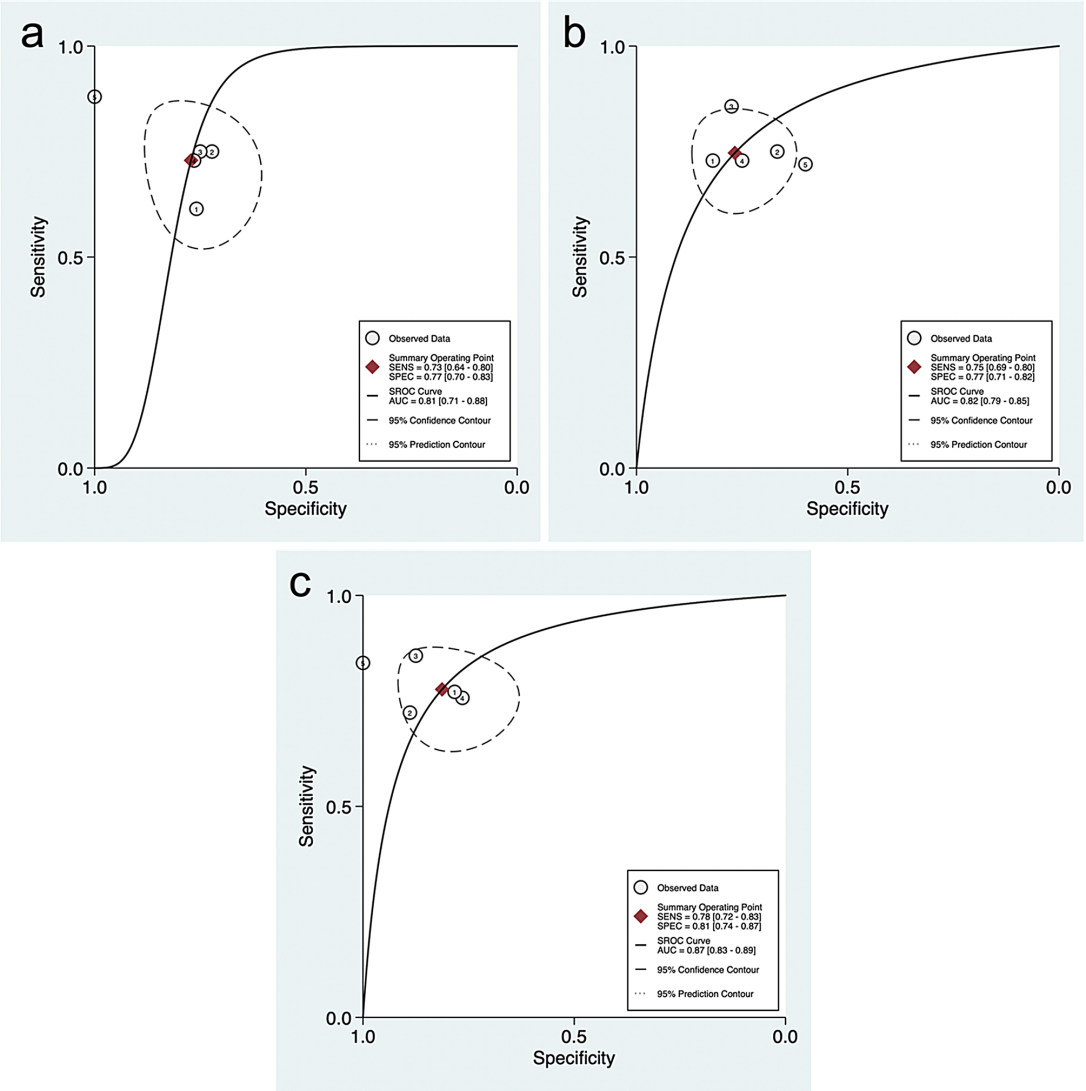
Summary receiver operating characteristic (SROC) curves for prediction of peritoneal metastasis in ovarian cancer. **a**) SROC curve of radiomics models. **b**) SROC curve of clinical models. **c**) SROC curve of combined (radiomics and clinical) models

As shown in Fig. 4, the pooled predictive performance of radiomics models showed an AUC of 0.81 (95% CI: 0.71–0.88). Clinical and clinical-radiomics models achieved an AUC of 0.82 (95% CI: 0.79–0.85), and 0.87 (95% CI: 0.83–0.89). Full results of pooled performance metrics are provided in Table 2.

**Table 2:** Pooled performance metrics from the Meta-Analysis

Model Parameter	Radiomics	Clinical	Combined (Radiomics + Clinical)
Area Under the Curve (AUC)	0.81 [0.71–0.88]	0.82 [0.79–0.85]	0.87 [0.83–0.89]
Sensitivity	0.73 [0.64–0.80]	0.75 [0.69–0.80]	0.78 [0.72–0.83]
Specificity	0.77 [0.70–0.83]	0.77 [0.71–0.82]	0.81 [0.74–0.87]
Positive Likelihood Ratio (PLR)	3.2 [2.3–4.4]	3.2 [2.5–4.1]	4.2 [2.9–5.9]
Negative Likelihood Ratio (NLR)	0.35 [0.25–0.49]	0.33 [0.26–0.42]	0.27 [0.21–0.36]
Diagnostic Odds Ratio (DOR)	9 [5–16]	10 [6–15]	15 [9–27]

All reported metrics are presented with their corresponding 95% Confidence Intervals [95% CI] in brackets

#### Heterogeneity analysis

**Fig. 5:**
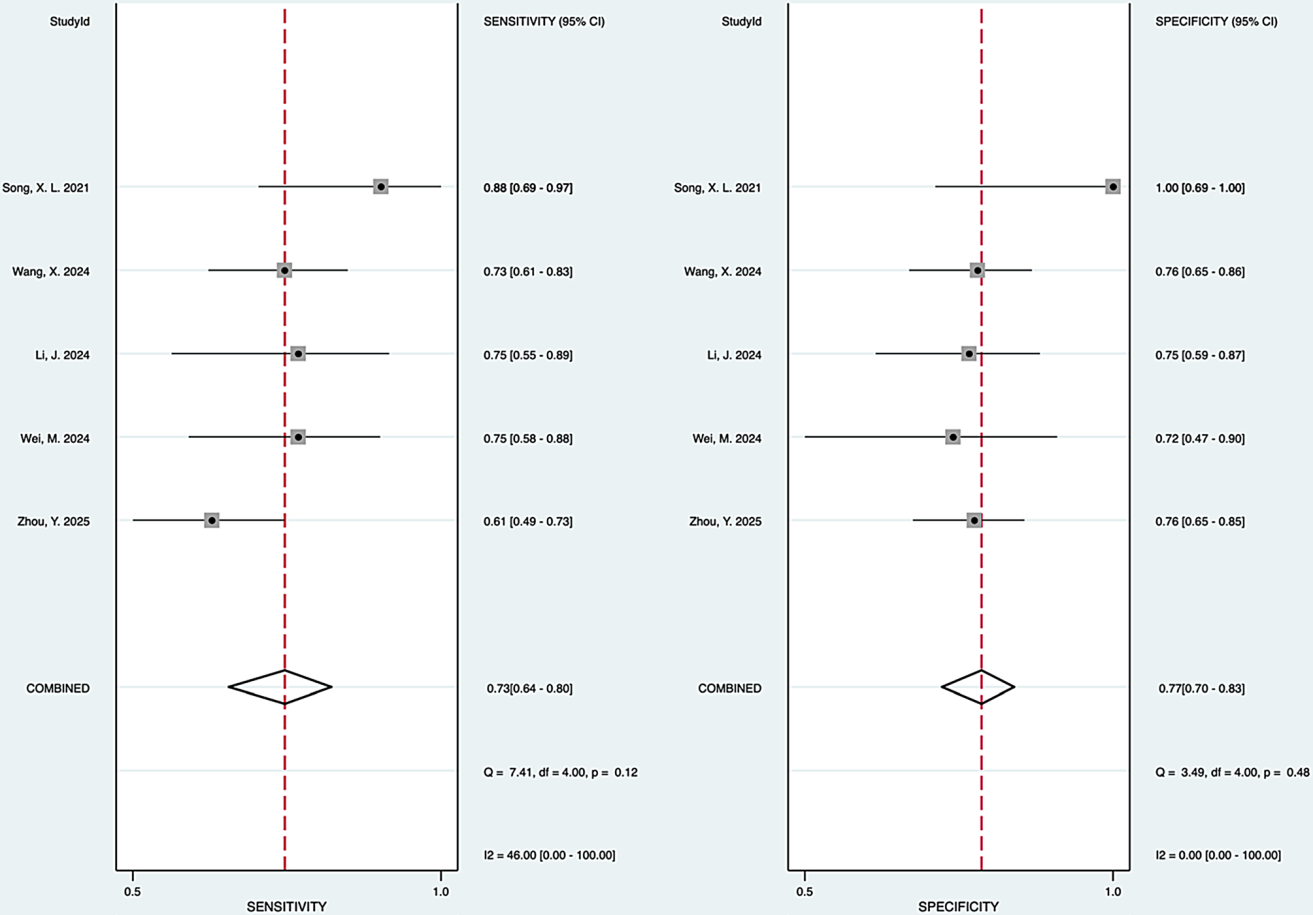
Forest plots of pooled sensitivity and specificity of studies included in the meta-analysis

The Forest plot of the pooled sensitivity showed an I² value of 46.00 (95%CI: 0.00-100.00) with a p-value of 0.12, which indicates no significant variability in results. The pooled specificity also showed an I² value of 0.00 (95%CI: 0.00-100.00), with a non-significant p-value of 0.48. Altogether, results show homogeneous pooled sensitivity and specificity (Fig. 5).

#### Subgroup analysis

Subgroup analysis revealed that study design, imaging modality, segmentation software, validation type, and choice of ML algorithm contributed to variability in predictive performance across studies. Studies conducted in single-center settings achieved higher pooled sensitivity (0.81 vs. 0.69, *p* < 0.01) and specificity (0.80 vs. 0.76, *p* = 0.02) than those from multi-center designs. In terms of segmentation tools, studies utilizing ITK-SNAP yielded superior performance compared to those using 3D Slicer, which corresponded with higher pooled sensitivity (0.76 vs. 0.65, *p* < 0.01), and specificity (0.78 vs. 0.76, *p* = 0.01) estimates. Validation strategy also influenced predictive metrics, as internally validated models outperformed externally validated ones in both sensitivity (0.81 vs. 0.69, *p* < 0.01) and specificity (0.80 vs. 0.76, *p* = 0.02). Studies with a prospective design achieved higher pooled specificity (1.00 vs. 0.76, *p* < 0.01) compared to retrospective studies. In addition, studies with training sample sizes greater than 250 reported lower sensitivity (0.66 vs. 0.76, *p* < 0.01) and lower specificity (0.75 vs. 0.78, *p* < 0.01) compared with those with smaller sample sizes. Studies with a METRICS score above 80%, categorized as excellent quality, demonstrated lower sensitivity (0.69 vs. 0.81, *p* < 0.01) and lower specificity (0.76 vs. 0.80, *p* = 0.02) compared with studies of lower quality. Furthermore, studies with a high risk of bias showed lower sensitivity (0.66 vs. 0.76, *p* < 0.01) and lower specificity (0.75 vs. 0.78, *p* < 0.01) compared with those assessed as low risk of bias. Full results from subgroup analysis are provided in Table 3.

**Table 3:** Findings from subgroup analysis

Parameter	Category	N	Sensitivity	P1	Specificity	P2	Joint Model Analysis				
							LRTChi2	p-value	I2	I2lo	I2hi
Design	Multi-center	3	0.69 [0.62–0.76]	< 0.01	0.76 [0.69–0.82]	0.02	3.04	0.22	34	0	100
	Single-center	2	0.81 [0.71–0.92]		0.80 [0.69–0.91]						
	Prospective	1	0.88 [0.75–1.00]	0.49	1.00 [1.00–1.00]	< 0.01	9.12	0.01	78	52	100
	Retrospective	4	0.70 [0.63–0.76]		0.76 [0.70–0.81]						
Modality	MRI	3	0.76 [0.69–0.84]	0.33	0.78 [0.70–0.86]	0.03	2.91	0.23	31	0	100
	Other	2	0.65 [0.56–0.75]		0.76 [0.68–0.83]						
Segmentation Software	3D Slicer	2	0.65 [0.56–0.75]	< 0.01	0.76 [0.68–0.83]	0.01	2.91	0.23	31	0	100
	ITK-SNAP	3	0.76 [0.69–0.84]		0.78 [0.70–0.86]						
Validation	External	3	0.69 [0.62–0.76]	< 0.01	0.76 [0.69–0.82]	0.02	3.04	0.22	34	0	100
	Internal	2	0.81 [0.71–0.92]		0.80 [0.69–0.91]						
ML Algorithm	WLC	2	0.65 [0.56–0.75]	< 0.01	0.76 [0.68–0.83]	0.01	2.91	0.23	31	0	100
	Other	3	0.76 [0.69–0.84]		0.78 [0.70–0.86]						
	SVM	2	0.74 [0.63–0.85]	0.2	0.76 [0.66–0.85]	0.02	0.29	0.87	0	0	100
	Other	3	0.72 [0.60–0.84]		0.78 [0.70–0.87]						
Train Size	> 250	2	0.66 [0.57–0.75]	< 0.01	0.75 [0.67–0.84]	< 0.01	2.62	0.27	24	0	100
	< 250	3	0.76 [0.69–0.84]		0.78 [0.70–0.85]						
METRICS	> 80%	3	0.69 [0.62–0.76]	< 0.01	0.76 [0.69–0.82]	0.02	3.04	0.22	34	0	100
	< 80%	2	0.81 [0.71–0.92]		0.80 [0.69–0.91]						
Risk of Bias	High	2	0.66 [0.57–0.75]	< 0.01	0.75 [0.67–0.84]	< 0.01	2.62	0.27	24	0	100
	Low	3	0.76 [0.69–0.84]		0.78 [0.70–0.85]						

ML: Machine Learning, N: Number of Studies, P1: p-value for sensitivity, P2: p-value for specificity, WLC: Weighed Linear Combination, SVM: Support Vector Machine, all reported metrics are presented with their corresponding 95% Confidence Intervals [95% CI] in brackets

#### Sensitivity analysis

Sensitivity analysis revealed that no study had a high influence or was identified as an outlier (Fig. S1).

#### Clinical utility and publication bias

**Fig. 6:**
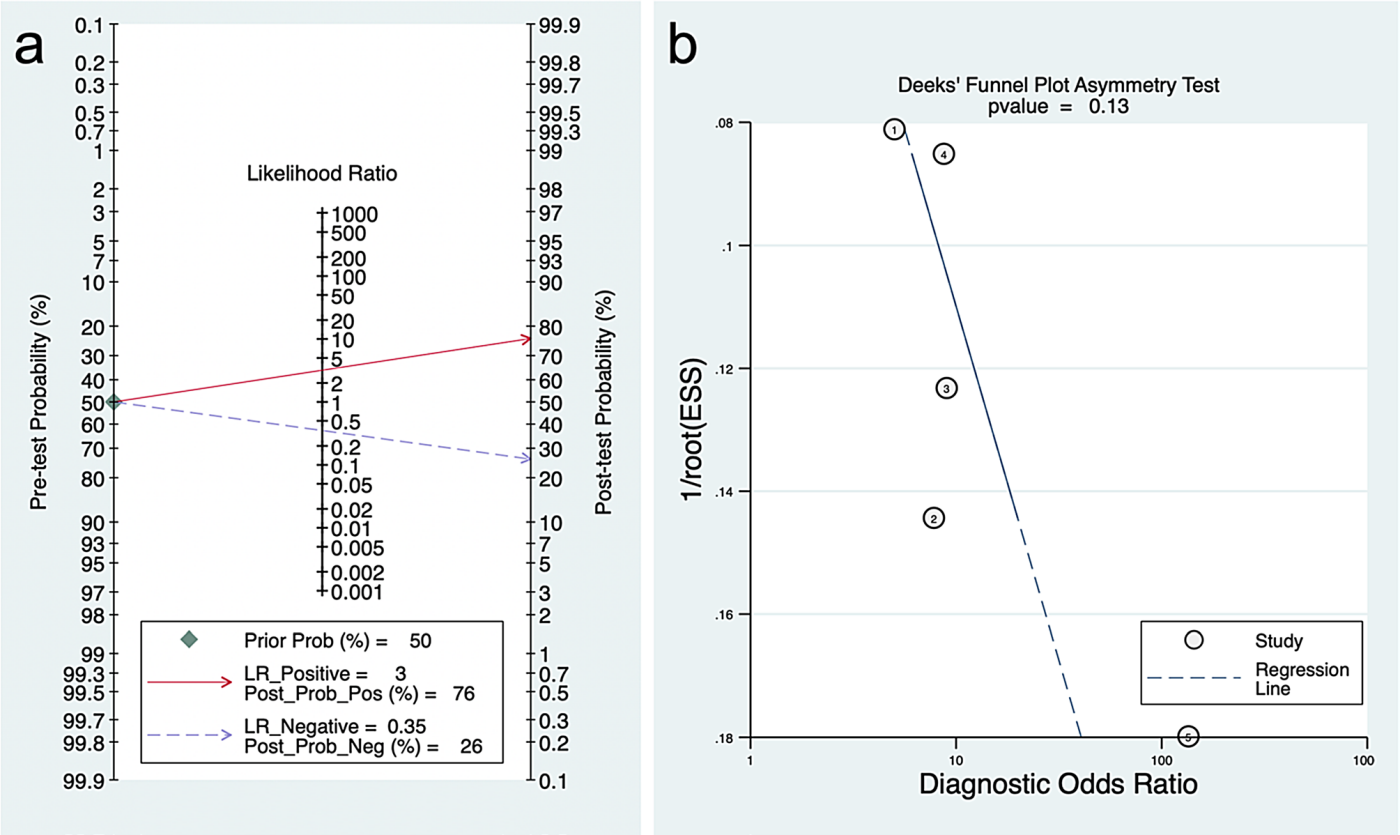
(**a**) Assessment of clinical utility of radiomics models for prediction of peritoneal metastasis in ovarian cancer using fagan plot. (**b**) Deeks funnel plot showing no publication bias (*p* = 0.13)

The Fagan plot demonstrated that, assuming a pre-test probability of 50%, the use of the model increased the post-test probability to 76% following a positive test result, corresponding to a positive likelihood ratio (PLR) of 3. In contrast, after a negative test result, the post-test probability decreased to 26%, yielding a negative likelihood ratio (NLR) of 0.35 (Fig. 6a).

#### Publication bias

The Deeks’ funnel plot asymmetry test did not indicate significant publication bias among the included studies (*p* = 0.13), supporting the reliability of the pooled diagnostic estimates (Fig. 6b).

## Discussion

In this systematic review and meta-analysis, we examined the diagnostic performance of radiomics-based ML models for predicting PM in OC. The meta-analysis results showed an acceptable AUC of 0.81, with a sensitivity of 0.73 and specificity of 0.77. While the AUC is not exceptionally high, it is considered acceptable given that we only included results from validation cohorts, which improves generalizability and robustness. To the best of our knowledge, we did not find any previous systematic reviews addressing this specific topic, which highlights the novelty of our work.

Currently, the management of OC with PM involves a multidisciplinary strategy guided by disease extent and patient-specific factors. Systemic chemotherapy remains a foundational component of treatment, often integrated with surgical intervention depending on the feasibility of achieving optimal cytoreduction. Initial evaluation through imaging and clinical assessment determines whether patients are candidates for primary surgery or would benefit from neoadjuvant chemotherapy to reduce tumor burden before operative management. The primary objective is to maximize oncologic outcomes by reducing residual disease while maintaining an acceptable safety profile. In selected cases, targeted therapies may be incorporated to further enhance treatment efficacy [32, 33].

At present, pathological evaluation of surgically resected specimens remains the definitive standard for diagnosing PM; however, this confirmation is only available postoperatively [34]. Preoperative imaging modalities such as CT and MRI, despite being routinely employed, exhibit significant limitations in sensitivity and accuracy, particularly in detecting small-volume or diffuse peritoneal disease. Additionally, these traditional imaging techniques are highly dependent on the reader’s expertise and are subject to considerable observer variability, which further compromises diagnostic consistency and reliability. Consequently, conventional imaging often fails to provide sufficient detail for reliable diagnosis, hindering optimal preoperative assessment and treatment planning [31].

In contrast, radiomics-based ML approaches hold considerable promise by extracting quantitative imaging features that capture subtle tumor heterogeneity beyond the capabilities of standard imaging interpretation. In contrast, radiomics-based ML approaches hold considerable promise by extracting quantitative imaging features that capture subtle tumor heterogeneity beyond the capabilities of standard imaging interpretation. Within the context of predicting PM in OC, radiomics enables enhanced preoperative detection of metastatic involvement that often remains occult on conventional imaging modalities such as CT and MRI. By providing a non-invasive, objective, and reproducible analysis of routine imaging data, radiomics addresses the inherent limitations of traditional techniques, including their dependence on reader expertise and significant interobserver variability. The high-dimensional features derived through radiomics facilitate comprehensive characterization of peritoneal lesions, improving the sensitivity and specificity of metastasis prediction. This capability is critical for accurate risk stratification and personalized treatment planning, allowing clinicians to tailor surgical and therapeutic approaches more effectively [35]. Furthermore, radiomics has the potential to minimize unnecessary exploratory surgeries by better delineating metastatic burden before intervention. Its standardized quantitative metrics also offer promise for monitoring disease progression and therapeutic response over time [36]. Importantly, integrating radiomics-derived features with clinical and molecular data may further enhance predictive accuracy, supporting more precise and individualized management strategies in OC patients at risk for PM.

The promising diagnostic accuracy of radiomics-based ML models for predicting PM in OC has important potential clinical implications. By enabling more accurate and non-invasive preoperative detection of metastatic involvement, these models could guide clinicians in optimizing treatment strategies—such as selecting candidates for primary cytoreductive surgery versus neoadjuvant chemotherapy—thereby improving surgical planning and patient outcomes. Enhanced risk stratification may also reduce unnecessary exploratory surgeries and associated morbidities, while supporting personalized treatment decisions tailored to metastatic burden. Moreover, radiomics could facilitate longitudinal monitoring of disease progression and therapeutic response, ultimately contributing to more precise and adaptive management of OC patients.

Despite these advantages, the integration of radiomics into clinical workflow requires careful consideration. Translation from research settings to daily practice demands standardized imaging protocols, harmonized feature extraction methods, and robust external validation across diverse populations. Automated pipelines capable of seamlessly processing routine imaging data and generating clinically interpretable outputs are essential for adoption. In addition, clear guidelines on how radiomics results should be combined with established clinical and pathological indicators are needed to facilitate decision-making. Successful integration will also depend on interdisciplinary collaboration between radiologists, oncologists, surgeons, and data scientists to ensure that radiomics findings are not only technically reliable but also clinically actionable. Ultimately, embedding radiomics into existing diagnostic and treatment pathways could support more efficient triage, optimize treatment allocation, and advance precision oncology in ovarian cancer.

An important step toward evaluating the clinical value of radiomics-based models is the application of decision curve analysis (DCA). Unlike traditional performance metrics such as AUC, which quantify overall discrimination, Decision curve analysis is used to determine the clinical usefulness of a model by quantifying net benefit across different probability thresholds [37]. This approach allows for direct comparison of whether the use of a radiomics model would result in better decision-making compared with existing clinical strategies, such as operating on all patients or on none. By quantifying the balance between true positives and false positives in clinically relevant contexts, DCA provides insight into the practical implications of model implementation. A strong aspect of the included studies is that all of them reported decision curve analysis. In each study, the DCA of the radiomics and combined models demonstrated good performance over a broad spectrum of threshold probabilities, whereas the clinical models showed limitations at certain thresholds in some studies. Future research should continue to incorporate DCA to more comprehensively evaluate the clinical utility of predictive models.

We evaluated the methodological quality of the included studies using the METRICS tool to identify strengths and deficiencies. The overall quality scores ranged from 64.50% to 86.30%, with a mean of 77.62%, indicating generally acceptable methodological standards. However, several notable gaps were evident. Only one out of six studies explicitly adhered to radiomics- or AI-specific reporting guidelines such as TRIPOD [38] or CLAIM [39]. Adherence to these frameworks is vital for ensuring transparency, reproducibility, and clinical utility, particularly in high-dimensional ML-based diagnostic studies where overfitting and interpretability remain key concerns [40]. Additionally, three out of six studies were single-center, which limits the diversity of patient populations and imaging equipment. In contrast, multicenter designs enhance external validity and support generalization across varying clinical environments [41]. Regarding validation strategies, one study failed to perform any validation on an independent cohort, which significantly undermines confidence in its findings. Among the remaining five, three included external validation, which is a critical strength. External validation provides a more robust estimate of real-world performance and reduces the risk of inflated metrics from internal data reuse [42]. The most underdeveloped domain across all studies was open science. None of the studies publicly shared their raw data or trained models, and only one provided access to its code. This lack of transparency hampers reproducibility and limits opportunities for independent verification or further method development. Embracing open science practices—such as sharing annotated datasets, radiomics pipelines, and ML code via repositories like GitHub—is essential to advancing the field, facilitating collaboration, and building cumulative scientific knowledge.

Although our meta-analysis suggests that radiomics holds promise for predicting PM in OC, the current evidence base remains limited and warrants cautious interpretation. The results, while encouraging, require further validation to ensure robustness and generalizability across broader clinical contexts. The relatively small number of included studies, combined with methodological and population homogeneity, underscores the need for additional research involving more diverse patient cohorts, ideally through multicenter collaborations that reflect real-world variability in imaging protocols, equipment, and clinical practice. Furthermore, while the overall methodological quality of the studies was rated as high, consistent application of validated reporting standards and methodological frameworks must be emphasized to strengthen future work. Greater consistency in image acquisition, segmentation, feature extraction, and model validation are essential to reduce bias and improve reproducibility. Importantly, future studies should prioritize rigorous external validation using independent datasets and clearly defined endpoints to better assess model performance and clinical utility. As the field advances, more transparent and standardized workflows, along with adoption of open science principles, will be critical to solidify the clinical translation of radiomics in OC care.

Based on our subgroup analysis, studies employing a single-center design demonstrated higher pooled sensitivity (0.81 [0.71–0.92] vs. 0.69 [0.62–0.76], *p* = 0.00) and specificity (0.80 [0.69–0.91] vs. 0.76 [0.69–0.82], *p* = 0.02) compared to those using multicenter designs. Similarly, studies utilizing internal validation showed better diagnostic performance than those with external validation, with sensitivity of 0.81 [0.71–0.92] vs. 0.69 [0.62–0.76] (*p* = 0.00) and specificity of 0.80 [0.69–0.91] vs. 0.76 [0.69–0.82] (*p* = 0.02). Although the number of studies in each subgroup was limited, these findings suggest that higher performance in single-center or internally validated studies may be influenced by methodological biases such as overfitting, reduced data variability, or lack of generalizability. Importantly, strong performance in homogeneous settings does not necessarily translate to broader clinical utility. Similarly, studies with training sample sizes above 250 showed reduced sensitivity (0.66 [0.57–0.75] vs. 0.76 [0.69–0.84], *p* < 0.01) and specificity (0.75 [0.67–0.84] vs. 0.78 [0.70–0.85], *p* < 0.01) compared with studies that used smaller cohorts. This counterintuitive finding may reflect greater heterogeneity in larger datasets, which can challenge model stability and reduce apparent performance, and it also highlights the need for more standardized methods and robust external validation in future large-scale studies. Studies with a METRICS score above 80%, categorized as excellent quality, demonstrated lower sensitivity (0.69 [0.62–0.76] vs. 0.81 [0.71–0.92], *p* < 0.01) and lower specificity (0.76 [0.69–0.82] vs. 0.80 [0.69–0.91], *p* = 0.02) compared with studies of lower quality. This pattern likely reflects that higher quality studies apply stricter design, reporting, and validation standards, which reduce overfitting and produce more conservative but more reliable estimates of diagnostic accuracy. In contrast, subgroup analyses based on other covariates—such as imaging modality, or ML algorithm—were largely inconclusive or did not reach statistical significance.

To further evaluate predictive performance and explore potential clinical utility, we also included studies that developed ML models based on clinical features alone, as well as those that combined clinical features with radiomic data. Interestingly, clinical models demonstrated a slightly higher pooled AUC of 0.82 [95% CI: 0.79–0.85] compared to radiomics-only models, which had an AUC of 0.81 [95% CI: 0.71–0.88]. However, the overlap in confidence intervals suggests that this difference is not statistically significant. Notably, combined clinical–radiomics models yielded the highest pooled AUC at 0.87 [95% CI: 0.83–0.89], outperforming both individual model types. Nevertheless, due to the overlap in confidence intervals in these comparisons as well, the superiority of the combined models cannot be definitively confirmed. These findings underscore the complementary nature of clinical and radiomic information and suggest that integrative approaches may enhance predictive accuracy, although further validation in prospective studies is required.

Several clinically significant indicators are associated with the presence of PM and with survival prognosis in OC. Among them, cancer antigen 125 (CA-125) is one of the most widely used serum biomarkers [43]. Elevated CA-125 levels have been correlated with advanced disease stage, tumor burden, and the likelihood of peritoneal dissemination, and it is frequently employed to monitor treatment response and recurrence. Human epididymis protein 4 (HE4) is another well-established biomarker with demonstrated utility in distinguishing malignant from benign ovarian tumors [44]. When used alongside CA-125, HE4 improves diagnostic accuracy and has been linked to poorer prognoses and increased metastatic potential. Notably, these two clinical biomarkers—CA-125 and HE4—were among the most frequently incorporated features in the clinical models developed across the studies included in our analysis, highlighting their central role in predictive modeling for PM in OC. However, the clinical models also incorporated heterogeneous sets of variables across studies, which introduces methodological variability and should be considered a limitation of our analysis.

Our study has some limitations. First, the number of available studies was limited; however, this reflects the current state of the literature, highlighting the need for further research in this area. Second, one study was excluded from our meta-analysis due to the lack of a validation cohort, but this also strengthens our analysis as we only included studies with validation cohorts, enhancing the generalizability of our findings. This study is also limited by the pooling of radiomics models developed from different imaging modalities (CT, MRI, ultrasound). While all radiomic features share similar computational principles, modality-specific characteristics may influence performance. Because of the small number of available studies, we could not conduct fully separate meta-analyses and instead performed subgroup analyses by modality, which only partially addresses this limitation. Among the strengths of our study, low heterogeneity was observed across included studies, with no evidence of publication bias or sensitivity issues, which supports the robustness and reliability of our results.

Future studies should focus on including larger, more diverse populations through multicenter collaborations to enhance the generalizability and robustness of radiomics models. Prospective designs with standardized imaging protocols and thorough external validation are essential to confirm predictive performance across different clinical settings. Additionally, efforts toward open science (for example, sharing data and code), will improve transparency and reproducibility. Incorporating advanced imaging modalities and integrating multi-omics data could further refine prediction accuracy and clinical utility in the management of PM in OC.

## Conclusion

Radiomics-based ML models show potential for predicting PM in OC, offering a non-invasive and objective tool to complement traditional imaging. By improving preoperative risk assessment, these models have the potential to enhance personalized treatment planning, optimize surgical decision-making, and ultimately improve patient outcomes. However, further large-scale, multicenter, and prospective studies with standardized methodologies and external validation are essential to confirm their clinical utility and facilitate widespread adoption.

## Supplementary Information

Below is the link to the electronic supplementary material.

Supplementary Material 1

## Data Availability

This study did not involve the generation or analysis of any proprietary or confidential datasets; all data utilized are publicly available and presented in the tables and supplementary materials.

## Abbreviations

OC: Ovarian Cancer
PM: Peritoneal Metastasis
AUC: Area Under the Curve
ML: Machine Learning
SROC: Summary Receiver Operating Characteristic
