# Supplementary material for: Radiomics-based machine learning in the prediction of peritoneal metastasis in ovarian cancer: a systematic review and meta-analysis
Source: BMC Med Imaging. 2025 Dec 2;26:6. doi: 10.1186/s12880-025-02068-3 (PMC12777038; doi:10.1186/s12880-025-02068-3)
Supplement: Supplementary file 1 — Supplementary Material 1 [file 12880_2025_2068_MOESM1_ESM.docx]

**Table S1:** Full search strategy query across different databases.

| **Database** | **Search query** | **Results** |
| --- | --- | --- |
| PubMed | ("peritoneal metastas*"[Title/Abstract] OR "peritoneal carcinomatos*"[Title/Abstract] OR "peritoneal neoplasm*"[Title/Abstract])  AND  ("ovar*"[Title/Abstract])  AND  ("Artificial intelligence"[Title/Abstract] OR "nomogram"[Title/Abstract] OR "AI"[Title/Abstract] OR "AI-Based"[Title/Abstract] OR "AI-Driven"[Title/Abstract] OR "machine learning*"[Title/Abstract] OR "machine-learning*"[Title/Abstract] OR "radiomic*"[Title/Abstract] OR "textur*"[Title/Abstract] OR "deep learning*"[Title/Abstract] OR "deep-learning*"[Title/Abstract] OR "neural network*"[Title/Abstract] OR "transfer learning*"[Title/Abstract] OR "transfer-learning*"[Title/Abstract] OR "convolutional neural network*"[Title/Abstract] OR "CNN"[Title/Abstract]) | 21 |
| Scopus | TITLE-ABS-KEY ( "peritoneal metastas*" OR "peritoneal carcinomatos*" OR "Peritoneal Neoplasm*" )  AND  TITLE-ABS-KEY ( "ovar*" )  AND  TITLE-ABS-KEY ( ( "Artificial intelligence" OR "nomogram" OR "AI" OR "AI-Based" OR "AI-Driven" OR "machine learning*" OR "machine-learning*" OR "radiomic*" OR "textur*" OR "deep learning*" OR "deep-learning*" OR "neural network*" OR "transfer learning*" OR "transfer-learning*" OR "convolutional neural network*" OR "CNN" ) | 41 |
| Web of Science | TS=("peritoneal metastas*" OR "peritoneal carcinomatos*" OR "peritoneal neoplasm*")  AND  TS=("ovar*")  AND  TS=("Artificial intelligence" OR "nomogram" OR "AI" OR "AI-Based" OR "AI-Driven" OR "machine learning*" OR "machine-learning*" OR "radiomic*" OR "textur*" OR "deep learning*" OR "deep-learning*" OR "neural network*" OR "transfer learning*" OR "transfer-learning*" OR "convolutional neural network*" OR "CNN") | 49 |
| Embase | ("peritoneal metastas*":ti,ab OR "peritoneal carcinomatos*":ti,ab OR "peritoneal neoplasm*":ti,ab)  AND  ("ovar*":ti,ab)  AND  ("Artificial intelligence":ti,ab OR "nomogram":ti,ab OR "AI":ti,ab OR "AI-Based":ti,ab OR "AI-Driven":ti,ab OR "machine learning*":ti,ab OR "machine-learning*":ti,ab OR "radiomic*":ti,ab OR "textur*":ti,ab OR "deep learning*":ti,ab OR "deep-learning*":ti,ab OR "neural network*":ti,ab OR "transfer learning*":ti,ab OR "transfer-learning*":ti,ab OR "convolutional neural network*":ti,ab OR "CNN":ti,ab) | 30 |

**Table S2:** QUADAS-2 tailored signaling questions in four domains.

| **Domain** | **Item** | **Question** | **Question number** |
| --- | --- | --- | --- |
| Patient Selection (D1) | Signaling Questions | Was the study's start and end point clearly defined, and were patients enrolled consecutively or randomly? | Q1 |
|  |  | Were the inclusion and exclusion criteria clearly defined, and were they applied consistently to all participants? | Q2 |
|  |  | Did the study perform reasonable and justified exclusions? | Q3 |
|  | Risk of Bias | Could the selection of patients have introduced bias? | ROB D1 |
|  | Applicability Concern | Are there concerns that the included patients do not match the review question? | Applicability D1 |
| Index Test (D2) | Signaling Questions | Was the segmentation and preprocessing of imaging data performed without knowledge of the reference test results? | Q4 |
|  |  | Was a consistent imaging protocol (e.g., imaging sequence, parameters) used for all patients? If not, was any task such as standardization, harmonization, or robust feature selection performed to address that? | Q5 |
|  |  | Was segmentation validation conducted using intraobserver or interobserver reproducibility analysis, or by performing test-retest tasks? | Q6 |
|  | Risk of Bias | Could the conduct or interpretation of the index test have introduced bias? | ROB D2 |
|  | Applicability Concern | Are there concerns that the index test, its conduct, or interpretation differ from the review question? | Applicability D2 |
| Reference Standard (D3) | Signaling Questions | Did the study employ a reliable reference standard to correctly classify the target outcome? | Q7 |
|  |  | Were the reference standard results interpreted in a blinded manner, without knowledge of the index test results? | Q8 |
|  | Risk of Bias | Could the reference standard, its conduct, or its interpretation have introduced bias? | ROB D3 |
|  | Applicability Concern | Are there concerns that the target condition as defined by the reference standard does not match the review question? | Applicability D3 |
| Flow and Timing (D4) | Signaling Questions | Was a reference standard applied to all patients? | Q9 |
|  |  | Was the same reference standard used for all patients? | Q10 |
|  |  | Was a suitable time interval present between the reference standard and the index test? Was it within the same reasonable range for all patients? | Q11 |
|  | Risk of Bias | Could the patient flow have introduced bias? | ROB D4 |

**Table S3:** Full answers to the QUADAS-2 assessments of risk of bias and applicability concerns.

| Study | Q1 | Q2 | Q3 | ROB D1 | Applicability D1 | Q4 | Q5 | Q6 | ROB D2 | Applicability D2 | Q7 | Q8 | ROB D3 | Applicability D3 | Q9 | Q10 | Q11 | ROB D4 |
| --- | --- | --- | --- | --- | --- | --- | --- | --- | --- | --- | --- | --- | --- | --- | --- | --- | --- | --- |
| Zhou, Y. 2025 | No | Yes | Yes | High | Low | Unclear | Yes | Yes | Unclear | Low | Yes | Yes | Low | Low | Yes | Yes | Yes | Low |
| Wei, M. 2024 | Yes | Yes | Yes | Low | Low | Yes | Yes | No | High | Low | Yes | Yes | Low | Low | Yes | Yes | Yes | Low |
| Li, J. 2024 | Yes | Yes | Yes | Low | Low | Yes | Yes | Yes | Low | Low | Yes | Yes | Low | Low | Yes | Yes | Yes | Low |
| Wang, X. 2024 | Yes | Yes | Yes | Low | Low | Yes | Yes | Yes | Low | Low | Yes | Yes | Low | Low | Yes | Yes | Yes | Low |
| Yu, X. Y. 2021 | Yes | Yes | Yes | Low | Low | Yes | Yes | Yes | Low | Low | Yes | Yes | Low | Low | Yes | Yes | Yes | Low |
| Song, X. L. 2021 | Yes | Yes | Yes | Low | Low | Yes | Yes | Yes | Low | Low | Yes | Yes | Low | Low | Yes | Yes | Yes | Low |

**Table S4:** METRICS tool answers and evaluation of methodological quality across studies.

| Items/Conditions | Definitions | Weights | Zhou, Y. 2025 | Wei, M. 2024 | Li, J. 2024 | Wang, X. 2024 | Yu, X. Y. 2021 | Song, X. L. 2021 |
| --- | --- | --- | --- | --- | --- | --- | --- | --- |
| Study Design |  |  |  |  |  |  |  |  |
| Item#1 | ? Adherence to radiomics and/or machine learning-specific checklists or guidelines | 0.0368 | no | yes | no | no | no | no |
| Item#2 | ? Eligibility criteria that describe a representative study population | 0.0735 | yes | yes | yes | yes | yes | yes |
| Item#3 | ? High-quality reference standard with a clear definition | 0.0919 | yes | yes | yes | yes | yes | yes |
| Imaging Data |  |  |  |  |  |  |  |  |
| Item#4 | ? Multi-center | 0.0438 | yes | yes | no | yes | no | no |
| Item#5 | ? Clinical translatability of the imaging data source for radiomics analysis | 0.0292 | yes | yes | yes | yes | yes | yes |
| Item#6 | ? Imaging protocol with acquisition parameters | 0.0438 | no | yes | yes | yes | yes | yes |
| Item#7 | ? The interval between imaging used and reference standard | 0.0292 | yes | yes | yes | yes | yes | yes |
| SegmentationC |  |  |  |  |  |  |  |  |
| Condition#1 | ? Does the study include segmentation? |  | yes | yes | yes | yes | yes | yes |
| Condition#2 | ? Does the study include fully automated segmentation? |  | no | no | no | no | no | no |
| Item#8 | ? Transparent description of segmentation methodology | 0.0337 | yes | yes | yes | yes | yes | yes |
| Item#9 | ? Formal evaluation of fully automated segmentationC | 0.0225 | n/a | n/a | n/a | n/a | n/a | n/a |
| Item#10 | ? Test set segmentation masks produced by a single reader or automated tool | 0.0112 | yes | yes | yes | yes | yes | yes |
| Image Processing and Feature Extraction |  |  |  |  |  |  |  |  |
| Condition#3 | ? Does the study include hand-crafted feature extraction? |  | yes | yes | yes | yes | yes | yes |
| Item#11 | ? Appropriate use of image preprocessing techniques with transparent description | 0.0622 | yes | yes | yes | yes | yes | yes |
| Item#12 | ? Use of standardized feature extraction softwareC | 0.0311 | yes | yes | yes | yes | yes | yes |
| Item#13 | ? Transparent reporting of feature extraction parameters, otherwise providing a default configuration statement | 0.0415 | no | no | yes | no | no | no |
| Feature Processing |  |  |  |  |  |  |  |  |
| Condition#4 | ? Does the study include tabular data? |  | yes | yes | yes | yes | yes | yes |
| Condition#5 | ? Does the study include end-to-end deep learning? |  | no | no | no | no | no | no |
| Item#14 | ? Removal of non-robust featuresC | 0.0200 | yes | no | yes | yes | yes | yes |
| Item#15 | ? Removal of redundant featuresC | 0.0200 | yes | yes | yes | yes | yes | yes |
| Item#16 | ? Appropriateness of dimensionality compared to data sizeC | 0.0300 | yes | yes | yes | yes | yes | yes |
| Item#17 | ? Robustness assessment of end-to-end deep learning pipelinesC | 0.0200 | n/a | n/a | n/a | n/a | n/a | n/a |
| Preparation for Modeling |  |  |  |  |  |  |  |  |
| Item#18 | ? Proper data partitioning process | 0.0599 | yes | yes | yes | yes | yes | yes |
| Item#19 | ? Handling of confounding factors | 0.0300 | yes | no | yes | no | no | no |
| Metrics and Comparison |  |  |  |  |  |  |  |  |
| Item#20 | ? Use of appropriate performance evaluation metrics for task | 0.0352 | yes | yes | yes | yes | no | yes |
| Item#21 | ? Consideration of uncertainty | 0.0234 | yes | yes | yes | yes | yes | yes |
| Item#22 | ? Calibration assessment | 0.0176 | yes | yes | yes | yes | yes | yes |
| Item#23 | ? Use of uni-parametric imaging or proof of its inferiority | 0.0117 | yes | yes | yes | yes | yes | no |
| Item#24 | ? Comparison with a non-radiomic approach or proof of added clinical value | 0.0293 | yes | yes | yes | yes | yes | yes |
| Item#25 | ? Comparison with simple or classical statistical models | 0.0176 | no | no | no | no | no | no |
| Testing |  |  |  |  |  |  |  |  |
| Item#26 | ? Internal testing | 0.0375 | yes | yes | yes | yes | no | yes |
| Item#27 | ? External testing | 0.0749 | yes | yes | no | yes | no | no |
| Open Science |  |  |  |  |  |  |  |  |
| Item#28 | ? Data availability | 0.0075 | no | no | no | no | no | no |
| Item#29 | ? Code availability | 0.0075 | no | no | yes | no | no | no |
| Item#30 | ? Model availability | 0.0075 | no | no | no | no | no | no |
| Total METRICS score: |  |  | 83.1% | 86.3% | 76.4% | 84.5% | 64.5% | 70.9% |
| ? Quality category: |  |  | Excellent | Excellent | Good | Excellent | Good | Good |

**Table S5:** 2×2 confusion matrices of clinical, radiomics, and combined models across the included studies.

| Study | Combined | | | | Clinical | | | | Combined | | | |
| --- | --- | --- | --- | --- | --- | --- | --- | --- | --- | --- | --- | --- |
|  | tp | fp | fn | tn | tp | fp | fn | tn | tp | fp | fn | tn |
| Zhou, Y. 2025 | 43 | 20 | 27 | 63 | 51 | 15 | 19 | 68 | 54 | 18 | 16 | 65 |
| Wei, M. 2024 | 27 | 5 | 9 | 13 | 27 | 6 | 9 | 12 | 26 | 2 | 10 | 16 |
| Li, J. 2024 | 21 | 10 | 7 | 30 | 24 | 9 | 4 | 31 | 24 | 5 | 4 | 35 |
| Wang, X. 2024 | 51 | 16 | 19 | 52 | 51 | 17 | 19 | 51 | 53 | 16 | 17 | 52 |
| Song, X. L. 2021 | 22 | 0 | 3 | 10 | 18 | 4 | 7 | 6 | 21 | 0 | 4 | 10 |


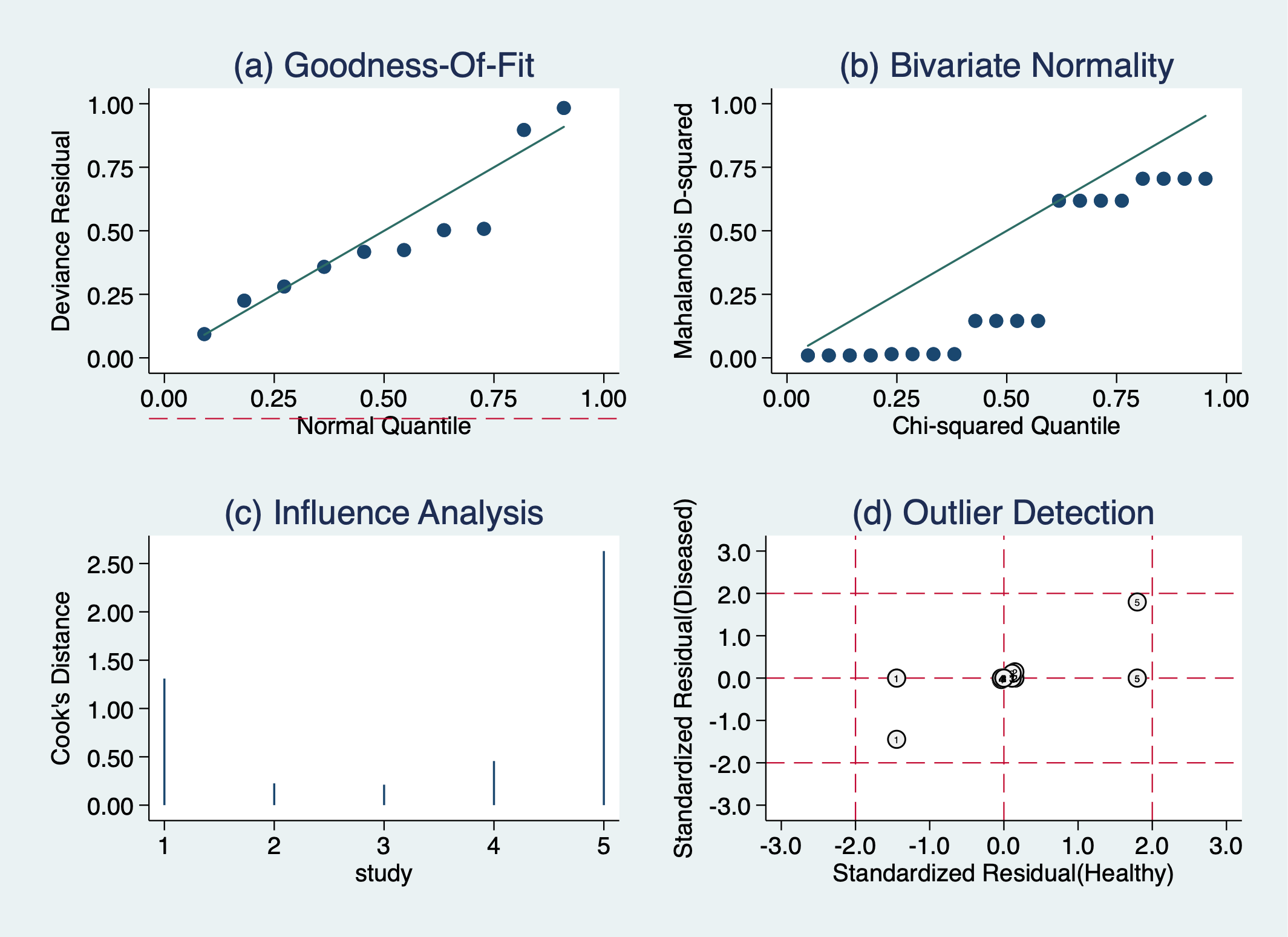


**Fig. S1:** Sensitivity Analysis: (a) Goodness of Fit Assessment, (b) Bivariate Normality Evaluation, (c) Influence Analysis, and (d) Outlier Detection.


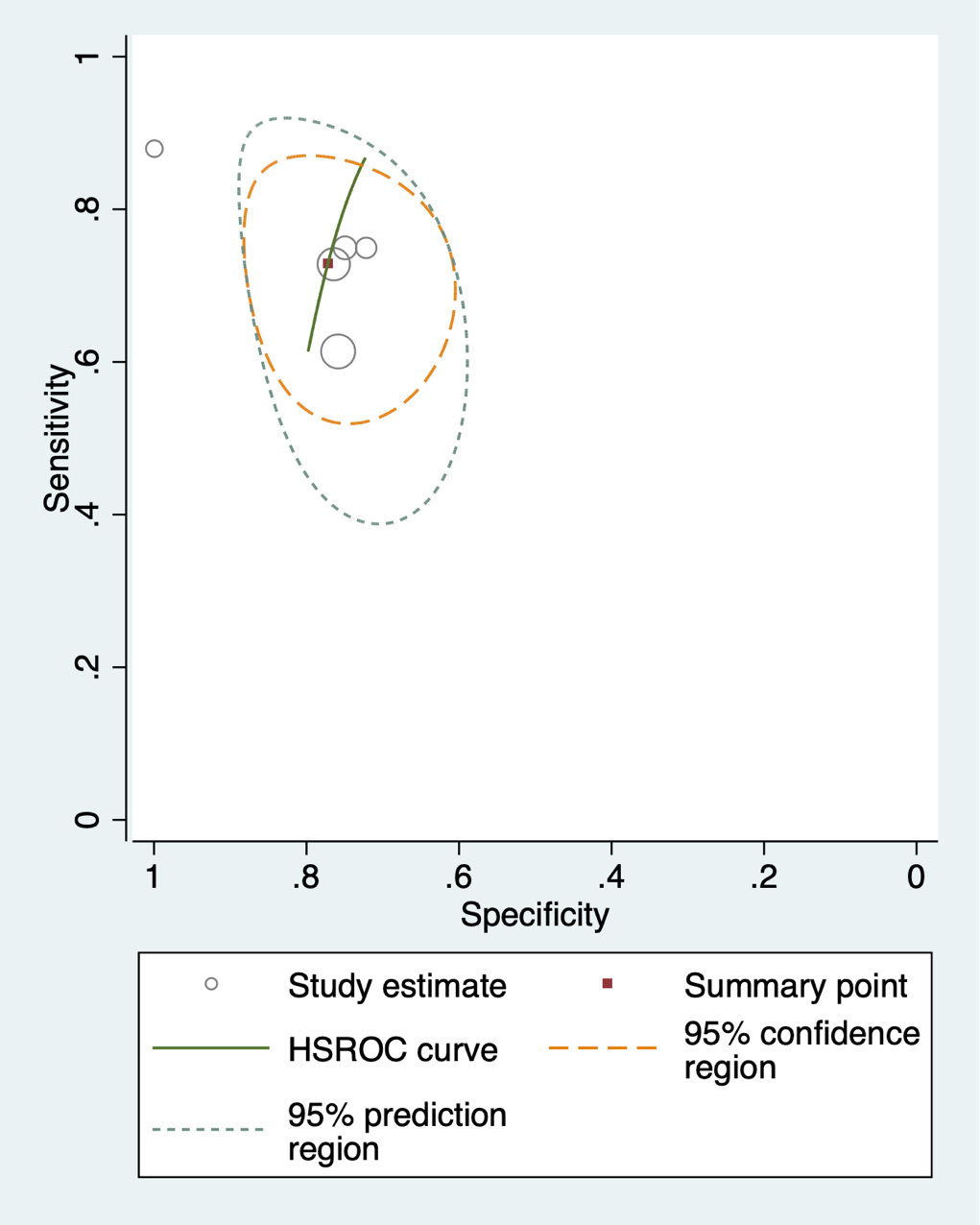


**Fig. S2:** Hierarchical summary receiver operating characteristic (HSROC) curve of radiomics models for preoperative prediction of peritoneal metastasis in ovarian cancer.

| **Section/topic** | **#** | **PRISMA-DTA Checklist Item** | **Reported on page #** |
| --- | --- | --- | --- |
| **TITLE / ABSTRACT** | | |  |
| Title | 1 | Identify the report as a systematic review (+/- meta-analysis) of diagnostic test accuracy (DTA) studies. | 2 |
| Abstract | 2 | Abstract: See PRISMA-DTA for abstracts. | Applied. |
| **INTRODUCTION** | | |  |
| Rationale | 3 | Describe the rationale for the review in the context of what is already known. | 3-4 |
| Clinical role of index test | D1 | State the scientific and clinical background, including the intended use and clinical role of the index test, and if applicable, the rationale for minimally acceptable test accuracy (or minimum difference in accuracy for comparative design). | 3-4 |
| Objectives | 4 | Provide an explicit statement of question(s) being addressed in terms of participants, index test(s), and target condition(s). | 5 |
| **METHODS** | | |  |
| Protocol and registration | 5 | Indicate if a review protocol exists, if and where it can be accessed (e.g., Web address), and, if available, provide registration information including registration number. | 5 |
| Eligibility criteria | 6 | Specify study characteristics (participants, setting, index test(s), reference standard(s), target condition(s), and study design) and report characteristics (e.g., years considered, language, publication status) used as criteria for eligibility, giving rationale. | 6 |
| Information sources | 7 | Describe all information sources (e.g., databases with dates of coverage, contact with study authors to identify additional studies) in the search and date last searched. | 5-6 |
| Search | 8 | Present full search strategies for all electronic databases and other sources searched, including any limits used, such that they could be repeated. | Table S1 |
| Study selection | 9 | State the process for selecting studies (i.e., screening, eligibility, included in systematic review, and, if applicable, included in the meta-analysis). | 5-6 |
| Data collection process | 10 | Describe method of data extraction from reports (e.g., piloted forms, independently, in duplicate) and any processes for obtaining and confirming data from investigators. | 6-7 |
| Definitions for data extraction | 11 | Provide definitions used in data extraction and classifications of target condition(s), index test(s), reference standard(s) and other characteristics (e.g. study design, clinical setting). | 7 |
| Risk of bias and applicability | 12 | Describe methods used for assessing risk of bias in individual studies and concerns regarding the applicability to the review question. | 7-8 |
| Diagnostic accuracy measures | 13 | State the principal diagnostic accuracy measure(s) reported (e.g. sensitivity, specificity) and state the unit of assessment (e.g. per-patient, per-lesion). | 8 |
| Synthesis of results | 14 | Describe methods of handling data, combining results of studies and describing variability between studies. This could include, but is not limited to: a) handling of multiple definitions of target condition. b) handling of multiple thresholds of test positivity, c) handling multiple index test readers, d) handling of indeterminate test results, e) grouping and comparing tests, f) handling of different reference standards | 8-9 |

Page 1 of 2

| **Section/topic** | **#** | **PRISMA-DTA Checklist Item** | **Reported on page #** |
| --- | --- | --- | --- |
| Meta-analysis | D2 | Report the statistical methods used for meta-analyses, if performed. | 8-9 |
| Additional analyses | 16 | Describe methods of additional analyses (e.g., sensitivity or subgroup analyses, meta-regression), if done, indicating which were pre-specified. | 8-9 |
| **RESULTS** | | |  |
| Study selection | 17 | Provide numbers of studies screened, assessed for eligibility, included in the review (and included in meta-analysis, if applicable) with reasons for exclusions at each stage, ideally with a flow diagram. | 9, Fig. 1 |
| Study characteristics | 18 | For each included study provide citations and present key characteristics including: a) participant characteristics (presentation, prior testing), b) clinical setting, c) study design, d) target condition definition, e) index test, f) reference standard, g) sample size, h) funding sources | Tables 1&2 |
| Risk of bias and applicability | 19 | Present evaluation of risk of bias and concerns regarding applicability for each study. | 9-10, Fig. 2&3, Tables S3-4 |
| Results of individual studies | 20 | For each analysis in each study (e.g. unique combination of index test, reference standard, and positivity threshold) report 2x2 data (TP, FP, FN, TN) with estimates of diagnostic accuracy and confidence intervals, ideally with a forest or receiver operator characteristic (ROC) plot. | Table S5 |
| Synthesis of results | 21 | Describe test accuracy, including variability; if meta-analysis was done, include results and confidence intervals. | 10-11, Table 2 |
| Additional analysis | 23 | Give results of additional analyses, if done (e.g., sensitivity or subgroup analyses, meta-regression; analysis of index test: failure rates, proportion of inconclusive results, adverse events). | 10-11, Table 3 |
| **DISCUSSION** | | |  |
| Summary of evidence | 24 | Summarize the main findings including the strength of evidence. | 11-12 |
| Limitations | 25 | Discuss limitations from included studies (e.g. risk of bias and concerns regarding applicability) and from the review process (e.g. incomplete retrieval of identified research). | 12-13-15-18 |
| Conclusions | 26 | Provide a general interpretation of the results in the context of other evidence. Discuss implications for future research and clinical practice (e.g. the intended use and clinical role of the index test). | 17-18 |
| **FUNDING** | | |  |
| Funding | 27 | For the systematic review, describe the sources of funding and other support and the role of the funders. | NA |

*Adapted From:*  McInnes MDF, Moher D, Thombs BD, McGrath TA, Bossuyt PM, The PRISMA-DTA Group (2018). Preferred Reporting Items for a Systematic Review and Meta-analysis of Diagnostic Test Accuracy Studies: The PRISMA-DTA Statement. JAMA. 2018 Jan 23;319(4):388-396. doi: 10.1001/jama.2017.19163.

Page 2 of 2
